# Adolescent “Lean PCOS” Is Characterized by Higher Insulin Resistance and Adverse Adipokine Profile

**DOI:** 10.1210/clinem/dgaf606

**Published:** 2025-12-08

**Authors:** Rachel C Whooten, Sheryl L Rifas-Shiman, Izzuddin M Aris, Wei Perng, Jorge E Chavarro, Emily Oken, Marie-France Hivert

**Affiliations:** Division of Pediatric Endocrinology, Department of Pediatrics, Massachusetts General Hospital for Children, Boston, MA 02114, USA; Division of General Academic, Department of Pediatrics, Massachusetts General Hospital for Children, Boston, MA 02114, USA; Division of Chronic Disease Research Across the Life Course, Department of Population Medicine, Harvard Medical School and Harvard Pilgrim Health Care Institute, Boston, MA 02215, USA; Division of Chronic Disease Research Across the Life Course, Department of Population Medicine, Harvard Medical School and Harvard Pilgrim Health Care Institute, Boston, MA 02215, USA; Department of Epidemiology, Lifecourse Epidemiology of Adiposity and Diabetes Center, Colorado School of Public Health, University of Colorado Denver Anschutz Medical Campus, Aurora, CO 80045, USA; Department of Nutrition, T. H. Chan Harvard School of Public Health, Boston, MA 02115, USA; Channing Division of Network Medicine, Department of Medicine, Brigham and Women's Hospital and Harvard Medical School, Boston, MA 02115, USA; Division of Chronic Disease Research Across the Life Course, Department of Population Medicine, Harvard Medical School and Harvard Pilgrim Health Care Institute, Boston, MA 02215, USA; Department of Nutrition, T. H. Chan Harvard School of Public Health, Boston, MA 02115, USA; Division of Chronic Disease Research Across the Life Course, Department of Population Medicine, Harvard Medical School and Harvard Pilgrim Health Care Institute, Boston, MA 02215, USA; Diabetes Unit, Massachusetts General Hospital, Boston, MA 02114, USA

**Keywords:** polycystic ovary syndrome, adolescent, body mass index, BMI, insulin resistance, HOMA-IR, adiponectin-leptin ratio, cardiometabolic biomarkers

## Abstract

**Objective:**

Although polycystic ovary syndrome (PCOS) is associated with high body mass index (BMI), less is known about the cardiometabolic manifestations of PCOS without excess adiposity. Among female adolescents enrolled in the Project Viva longitudinal prebirth cohort, we characterized growth, adiposity, and cardiometabolic biomarkers among those with vs without PCOS, stratified by BMI category.

**Methods:**

We defined PCOS at the mid-teen visit (mean age 17.7 years) as self-reported diagnosis or oligo-anovulation with clinical/biochemical hyperandrogenism. We obtained anthropometric and dual x-ray absorptiometry measurements. Within each BMI category (≥85th percentile vs < 85th percentile), we used unadjusted linear regression to compare growth trajectories, adiposity, and cardiometabolic biomarkers among those with vs without PCOS. We used mixed effects models to visually represent estimated BMI and linear growth trajectories.

**Results:**

Among 358 females with data at the mid-teen visit, n = 51 (14%) participants met our criteria for PCOS. Among females with BMI <85th percentile, those with PCOS (n = 27) had earlier age at peak height velocity [β = -.57 years; 95% confidence interval (CI) −0.96, −0.18], higher Homeostatic Model Assessment of Insulin Resistance (β = .77, 95% CI 0.23, 1.30), and lower adiponectin-leptin ratio (β = −.35, 95% CI −0.65, −0.06) vs without PCOS. Females with BMI ≥85th percentile had similar biomarkers by PCOS status. Adiposity measures did not differ by PCOS status within either BMI category.

**Conclusion:**

Within this population-based cohort, adolescents with PCOS and BMI <85th percentile had greater insulin resistance and adipose tissue dysfunction vs without PCOS. PCOS-associated metabolic dysfunction exist even among adolescents with BMI <85th percentile.

Polycystic ovary syndrome (PCOS) is a common reproductive and endocrine condition, impacting approximately 10% to 15% of women (1). While the diagnostic criteria for PCOS include irregular menses, hyperandrogenism, and/or polycystic ovarian morphology (among adult women) while also excluding other potential diagnoses, the clinical manifestations of PCOS expand beyond these traits to include cardiometabolic, reproductive, and mental health comorbidities, as well as an association with obesity, that together impose a substantial public health burden (2). As a result, it is critical to increase our understanding of PCOS, especially within the adolescent years when the first signs of PCOS may become apparent.

There is a significant literature gap relating to the presentation of PCOS during adolescence. Some of this is due to the challenges of diagnosing during adolescence, as diagnostic criteria, such as irregular menses or clinical hyperandrogenism manifesting as acne, are broadly common during the pubertal transition (3). Despite these limitations, there is evidence that the cardiometabolic correlates of PCOS are present among adolescents, including greater prevalence of insulin resistance and type 2 diabetes, hepatic steatosis, hypertension, hyperlipidemia, and metabolic syndrome (4-7).

Although those with PCOS are more likely to have overweight and obesity, PCOS occurs across the body mass index (BMI) spectrum (8). While adiposity may worsen the clinical presentation of the cardiometabolic associations of PCOS (9), individuals with PCOS without excess body weight (termed “lean” PCOS within the literature) are also at increased risk for metabolic syndrome (10). Less research has been done specifically among adolescents examining whether the cardiometabolic associations of PCOS may vary with adiposity status, with existing studies mostly cross-sectional and relying on clinical samples (5, 6, 11). While some literature has described increased tempo of childhood weight gain in association with later PCOS and taller childhood stature in association with PCOS, no studies to our knowledge have examined whether these may be different among those with PCOS by adiposity status (12-15). This gap in the literature is notable, as identifying early risk markers of PCOS before its full presentation is a potential strategy for early identification of PCOS risk to potentially mitigate severity and progression.

We sought to examine (1) cardiometabolic biomarkers in mid-late adolescence among adolescents with vs without PCOS and (2) adiposity and linear growth trajectories from infancy through adolescence, stratified by BMI category (<85th vs ≥85th percentile for age). We approached these descriptive aims within a well-characterized prebirth cohort, Project Viva, in which we have previously observed an association between mid-childhood adiposity and increased odds of later PCOS (16). We hypothesized that individuals with PCOS would have an adverse metabolic profile compared to peers without PCOS, with more pronounced abnormalities among those with both PCOS and BMI ≥85th percentile. Additionally, we hypothesized that individuals with PCOS with both normal and high BMI would have different growth trajectories, with individuals with PCOS and BMI ≥85th percentile displaying accelerated weight gain vs peers without PCOS and BMI ≥85th percentile.

## Methods

We performed analyses of adolescents participating in Project Viva, a prospective study of maternal-child health throughout the life course. We recruited pregnant women at their initial prenatal visit within a multispecialty group in eastern Massachusetts (1999-2002). In-person study visits occurred during infancy, early and mid-childhood, and early and mid-teen years. Between visits, participants received annual mailed or online questionnaires. Mothers gave written informed consent at enrollment and postpartum in-person visits; adolescents provided informed assent beginning at age 12 and informed consent once 18 years or older. The institutional review board of Harvard Pilgrim Health Care approved all study protocols. For purposes of the current analyses, all outcomes and exposures were assessed at the mid-teen visit.

**Figure 1.**
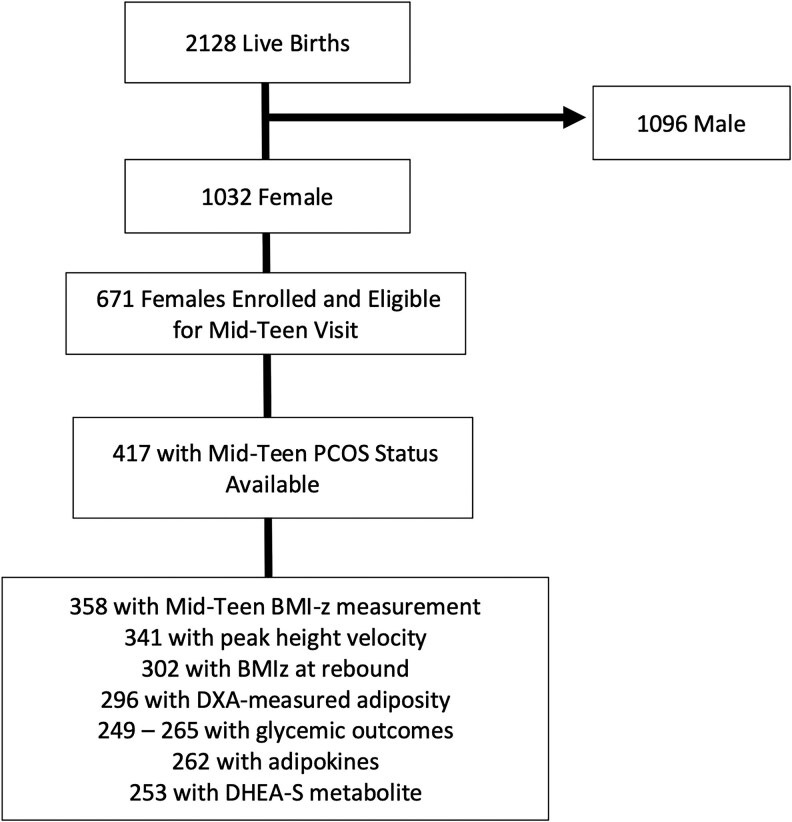
Flow chart of study sample.

Of 2128 live births (48% female), we included in this analysis all female participants with anthropometric measurements, biomarkers, and PCOS status available at the mid-teen visit (Fig. 1). Of 761 females still enrolled and eligible for a mid-teen visit, 417 completed the mid-teen questionnaire, and 358 had BMI-z measurement and blood available for biomarker assessment, forming the sample for this analysis. As noted in our prior work, those who were excluded from analyses with missing PCOS status at the mid-teen visit had lower mean birthweight for gestational age z-score and were less likely to have mothers who completed college (16).

### PCOS Classification

We classified probable PCOS in agreement with international evidence-based recommendations for diagnosing PCOS in adolescents, including the presence of (1) menstrual irregularities reflecting ovulatory dysfunction and (2) biochemical and/or clinical hyperandrogenism using methods described previously (16). In summary, we asked participants to report if they had a diagnosis of PCOS or were on an oral contraceptive to treat of PCOS. We classified participants as PCOS if they (1) reported PCOS diagnosis and/or (2) met criteria for probable PCOS via ovulatory dysfunction with biochemical and/or clinical hyperandrogenism. We assessed menstrual patterns on the mid-teen questionnaire and classified usual cycle length <21 days, > 35 days, or “too irregular to report” as well as primary amenorrhea as ovulatory dysfunction. We assessed clinical hyperandrogenism via self-report of hirsutism or acne using pictorial representations. We classified hirsutism as a simplified Ferriman-Gallwey (17) score of ≥3 (sum of 3 items scored 0-4, assessing terminal hair growth on upper/lower abdomen and chin) and moderate-severe acne as “several to many inflamed pustules, some containing pus or fluid.” For biochemical hyperandrogenism, we used plasma metabolite concentrations of dehydroepiandrosterone sulfate (DHEA-S) measured via untargeted plasma metabolomic profiling conducted by Metabolon Inc®. As total and/or free testosterone were not available within our cohort at this time, we chose DHEA-S given its clinical relevance and inclusion in evidence-based guidelines as an alternative measure when testosterone concentrations are not elevated (or not available) (2).

### Assessment of Adiposity and Growth

We obtained birthweight from the hospital medical records and calculated sex-specific z-scores based on weight for gestational age (18). Trained research assistants used a standardized protocol to measure participant height and weight at infancy (∼6 months), early childhood (∼3 years of age), mid-childhood (∼7 years of age), and early teen visits (∼13 years of age) with height, weight, and waist circumference mid-teen visit (∼17-20 years of age) (19). We calculated BMI based on weight (kg) divided by square of height (m^2^) and derived age- and sex-specific BMI percentiles using Centers for Disease Control and Prevention 2000 growth reference data (20). We calculated age at peak height velocity using previously described methods (21). We also calculated age at adiposity rebound and modeled BMI trajectories among participants with ≥2 BMI measurements from birth to 216 months of age using previously described methods (22, 23). At the mid-teen visit, participants completed whole-body dual radiograph absorptiometry (DXA) according to the manufacturer's recommendations (Hologic model Discovery A; Hologic, Bedford, MA). We calculated adiposity indices, including truncal fat mass and total fat mass percent.

### Measurement of Biomarkers

A phlebotomist obtained fasting blood specimens at the mid-teen visit (19). Insulin was measured via electrochemiluminescence immunoassay (catalog #12017547122, RRID:AB_2756877), glucose and lipid profile were measured enzymatically, high-sensitivity C-reactive protein was measured using immunoturbidimetric high-sensitivity assay (catalog #04628918190, RRID:AB_3668965), leptin was measured using ELISA (catalog #DLP00, RRID:AB_2783014), and adiponectin was measured using ELISA (catalog #DRP300, RRID:AB_2783020). We calculated adiponectin/leptin ratio as a marker of adipose tissue dysfunction and associated cardiometabolic risk (24). We used the Homeostatic Model Assessment of Insulin Resistance (HOMA-IR; glucose in mg/dL × insulin in μU/mL)/405) to calculate insulin resistance (25).

### Other Variables

We collected information on maternal characteristics at the time of enrollment, including self-reported height, prepregnancy weight, hypertension diagnosis and smoking status, and highest level of education. We classified maternal PCOS based on (1) International Classification of Diseases, Ninth Revision code for PCOS within the medical record at the time of index pregnancy or (2) self-report of diagnosis on the maternal questionnaire at the mid-late adolescent visit. Adolescents self-reported race and ethnicity on the age 19 questionnaire; if missing, we used maternal self-report of race and ethnicity or maternal report of child race and ethnicity from early childhood, as available. Adolescents also self-reported oral contraceptive usage on the mid-teen questionnaire. We collected age of participant's menarche based on first occurrence reported on age 9 through 16 questionnaires (maternal report) or mid-teen questionnaires (teen report).

### Analyses

We stratified all analyses by BMI category (<85th vs ≥85th percentile), with the cut-point of ≥85th percentile chosen based on the Centers for Disease Control and Prevention threshold for pediatric overweight and obesity (26). Within each stratum, we compared distributions of maternal and participant characteristics (Table 1), growth parameters (Table 2), and cardiometabolic biomarkers (Table 3) between participants with and without PCOS. For participant characteristics, we used *t*-tests for normally distributed continuous variables and chi-square for categorical variables. We used unadjusted linear regression to compare growth parameters and cardiometabolic markers. We conducted sensitivity analyses excluding adolescents classified as PCOS solely based on menstrual irregularities + acne criteria, given that acne is frequent in adolescence and difficult to assign specifically to PCOS. We calculated Spearman correlation coefficients to examine the associations between adipokines and BMI, stratified by PCOS status. We also created visual representations of estimated BMI and linear growth trajectories according to PCOS status and BMI stratification using mixed-effects models with natural cubic spline functions for age using previously described methods (22).

**Table 1.** **Project Viva participant characteristics according to presence of probable PCOS, stratified by BMI category at the mid-teen visit**.

	BMI <85th percentile (n = 267, 75%)			BMI ≥85th percentile (n = 91, 25%)		
	PCOS			PCOS		
	Absent	Present		Absent	Present	
	n = 240	n = 27	*P*-values	n = 67	n = 24	*P*-values
	Mean (SD) or n (%)	Mean (SD) or n (%)		Mean (SD) or n (%)	Mean (SD) or n (%)	
Maternal characteristics						
Prepregnancy BMI, kg/m^2^	23.4 (4.4)	23.8 (4.3)	0.68	26.6 (5.3)	30.0 (8.5)	.08
Prepregnancy BMI ≥30 kg/m^2^, n (%)	22 (9)	3 (11)	.74	18 (27)	10 (42)	.18
Maternal PCOS diagnosis*^a^*	8 (3)	2 (7)	.29	2 (3)	5 (21)	**.005**
Self-reported history of hypertension,*^a^* n (%)	14 (6)	2 (7)	.75	3 (4)	4 (17)	.**05**
Mother college graduate, n (%)	194 (81)	18 (67)	.08	39 (58)	13 (54)	.73
Pregnancy smoking status, n (%)*^a^*			.42			.73
Never	178 (74)	17 (63)		48 (72)	16 (67)	
Former	45 (19)	7 (26)		12 (18)	4 (17)	
Smoked during pregnancy	16 (7)	3 (11)		7 (10)	4 (17)	
Participant characteristics						
Child race and ethnicity, n (%)			.76			.19
Hispanic	13 (5)	2 (7)		13 (19)	7 (29)	
Non-Hispanic White	173 (72)	17 (63)		39 (58)	8 (33)	
Non-Hispanic Black	24 (10)	3 (11)		12 (18)	8 (33)	
Non-Hispanic Asian/other	30 (12)	5 (19)		3 (4)	1 (4)	
Age at mid-teen visit, years, mean (SD)	17.6 (0.6)	17.7 (0.7)	.45	17.7 (0.7)	17.7 (0.6)	.74
Age at first period, years, mean (SD)	13.0 (1.4)	12.5 (1.5)	.07	12.1 (1.1)	12.6 (1.5)	.12
OCP usage	47 (20)	8 (30)	.22	18 (27)	3 (13)	.15
Diagnostic criteria, n (%)						
Ovulatory dysfunction (irregular menses)	45 (19)	24 (89)	**<**.**0001**	9 (14)	21 (88)	**<**.**0001**
Hirsutism score ≥3	64 (27)	18 (69)	**<**.**0001**	19 (29)	18 (75)	**<**.**0001**
Moderate-severe acne	20 (8)	1 (4)	.40	4 (6)	3 (13)	.30
Top 25% DHEA-S	30 (18)	8 (44)	.**01**	8 (44)	9 (50)	.**003**
PCOS characteristics, n (%)						
Ovulatory dysfunction + clinical HA	−	18 (67)		—	19 (79)	
Ovulatory dysfunction + biochemical HA	—	7 (37)		—	9 (47)	
Self-reported PCOS diagnosis or OCP for PCOS	—	4 (15)		—	8 (33)	

Bold text indicates statistical significance with *P*-value <.05.

Abbreviations: BMI, body mass index; DHEA-S, dehydroepiandrosterone sulfate; HA, hyperandrogenism; OCP, oral contraceptive; PCOS, polycystic ovary syndrome.

^*a*^Maternal self-report of PCOS diagnosis, history of hypertension, and smoking status collected at enrollment.

**Table 2.** **Lifetime growth parameters by presence of probable PCOS, stratified by BMI category at mid-teen visit**.

	BMI <85th percentile					BMI ≥85th percentile				
	(n = 267, 75%)					(n = 91, 25%)				
	PCOS absent n = 240		PCOS present n = 27			PCOS absent n = 67		PCOS present n = 24		
	Mean	SD	Mean	SD	PCOS yes vs no	Mean	SD	Mean	SD	PCOS yes vs no
					β (95% CI)					β (95% CI)
Birth										
Birthweight for GA z-score	0.24	0.92	0.10	0.77	−.14 (−.50, .22)	0.32	1.02	0.22	0.81	−.10 (−.56, .36)
Adiposity—BMI rebound										
Age, months	64.1	19.5	58.3	21.4	−5.88 (−14.1, 2.29)	43.4	15.8	45.7	17.1	2.33 (−6.51,11.16)
BMI-z, units	−0.1	0.9	−0.2	0.7	−.06 (−.42, .30)	0.7	0.6	0.5	0.7	−.19 (−.55, .17)
Linear growth										
Age at peak height velocity, years	11.5	1.0	10.9	0.9	**−.57** (**−.96,−0.18)**	10.7	0.9	10.7	0.7	−.08 (−.50, 0.34)
Height at mid-teen visit, cm	165.3	6.4	165.2	5.5	−.11 (−2.62, 2.40)	164.5	6.0	163.6	7.1	−.87 (−3.84, 2.10)
Adiposity—mid-teen visit										
BMI, kg/m^2^	21.4	2.2	21.4	2.2	.01 (−.86, .88)	30.9	5.4	32.0	6.4	1.12 (−1.55, 3.79)
BMI-z, units	−0.02	0.75	−0.01	0.68	.01 (−.29, .30)	1.61	0.42	1.68	0.46	.06 (−.14, .27)
Waist circumference (cm)	74.6	6.8	73.8	6.0	−.81 (−3.48, 1.86)	95.3	13.4	96.4	13.6	1.13 (−5.25, 7.51)
DXA truncal fat mass, kg	7.1	2.4	7.0	1.7	−.13 (−1.17, .90)	15.8	5.2	18.1	6.8	2.24 (−.78, 5.26)
DXA total % fat	30.4	4.9	30.4	3.4	.02 (−2.09, 2.13)	40.7	4.5	41.6	5.8	.87 (−1.72, 3.46)

Bold text indicates statistical significance with *P*-value <.05.

Abbreviations: BMI, body mass index; CI, confidence interval; DXA, dual-energy X-ray absorptiometry; GA, gestational age; PCOS, polycystic ovary syndrome.

**Table 3.** **Mid-teen cardiometabolic biomarkers, according to presence of probable PCOS, stratified by BMI category at the mid-teen visit**.

	BMI <85th percentile					BMI ≥85th percentile				
	(n = 267, 75%)					(n = 91, 25%)				
	PCOS absent n = 240		PCOS present n = 27			PCOS absent n = 67		PCOS present n = 24		
	Mean	SD	Mean	SD	PCOS yes vs no	Mean	SD	Mean	SD	PCOS yes vs no
					β (95% CI)					β (95% CI)
Glycemia										
Fasting insulin, uU/mL	10.2	4.7	13.6	7.0	**3.41** (**.97, 5.84)**	17.3	9.3	14.9	6.6	−2.34 (−7.09, 2.41)
Fasting glucose, mg/dL	82.6	5.7	84.0	5.3	1.36 (−1.34, 4.07)	83.3	6.6	85.1	7.4	1.76 (−2.00, 5.51)
HOMA-IR, units	2.1	1.0	2.8	1.5	**.77** (**.23, 1.30)**	3.5	2.0	3.1	1.4	−.38 (−1.42, .67)
HbA1c, percent	5.1	0.3	5.1	0.3	.00 (−.13, .13)	5.1	0.4	5.1	0.3	−.03 (−.23, .18)
Lipids										
Total cholesterol, mg/dL	160.5	28.5	153.1	24.2	−7.47 (−21.2, 6.27)	164.1	30.5	165.3	31.4	1.26 (−15.5,17.98)
Triglycerides, mg/dL	64.9	24.5	61.9	22.6	−3.00 (−14.9, 8.87)	70.3	29.1	74.4	36.4	4.06 (−12.9,20.96)
HDL, mg/dL	60.9	12.6	58.9	9.2	−2.00 (−8.02, 4.02)	55.2	12.1	52.9	8.2	−2.26 (−8.39, 3.87)
Inflammatory markers and adipokines										
hsCRP, mg/L	0.8	1.5	0.8	0.9	−.03 (−.74, .68)	3.7	5.0	4.3	5.0	.64 (−2.08, 3.35)
Leptin, ng/mL	15.1	9.2	19.2	8.9	4.18 (−.30, 8.67)	45.4	24.3	47.3	27.0	1.92 (−11.7,15.51)
Adiponectin, ug/mL	8.4	3.3	7.3	3.0	−1.12 (−2.73, .50)	6.1	3.3	5.6	2.4	−.49 (−2.19, 1.20)
Adiponectin/leptin ratio	0.80	0.62	0.44	0.21	**−.35** (**−.65,−.06)**	0.18	0.14	0.18	0.21	.00 (−.09, .09)
Androgen concentration										
DHEA-S (metabolite)	−0.17	0.50	0.07	0.48	.24 (.00, .48)	−0.24	0.61	0.14	0.43	**.38** (**.07, .69)**

Bold text indicates statistical significance with *P*-value <.05.

Abbreviations: BMI, body mass index; CI, confidence interval; DHEA-S, dehydroepiandrosterone sulfate; HbA1c, hemoglobin A1c; HDL, high-density lipoprotein; HOMA-IR, Homeostatic Model Assessment of Insulin Resistance; hsCRP, high sensitivity C-reactive protein.

## Results

Participants were in majority non-Hispanic White (66%) with mothers who attained a college education (74%) (Table 1). Mean (SD) age at the time of the mid-teen visit was 17.7 (0.7) years. PCOS prevalence within the cohort was 14% (n = 51), of whom n = 27 (53%) had BMI <85th percentile and n = 24 (47%) BMI ≥85th percentile. Among those classified as having PCOS, self-report of PCOS diagnosis (clinical or use of oral contraceptive for PCOS treatment) was twice as frequent among those with BMI ≥ 85th percentile vs those with BMI <85th percentile (33% vs 15%).

In analyses stratified by BMI category (BMI ≥85th vs BMI <85th percentile), we compared females with vs without PCOS in each stratum. There was no difference in adiposity, including BMI, BMI z-score, waist circumference, or DXA measures among those with vs without PCOS (Table 2).

Among those with BMI ≥85th percentile, growth parameters including age at peak height velocity and age at BMI rebound as well as biomarkers related to markers of glycemia, lipids, inflammatory markers, and adipokines did not vary by PCOS status (Tables 2 and 3). However, among those with BMI <85th percentile, adolescents with PCOS vs those without had a younger mean (SD) age at peak height velocity [10.9 (0.9) vs 11.5 (1.0) years; β = −.57; 95% confidence interval (CI) −0.96, −0.18] and a trend toward a younger mean (SD) age at BMI rebound [58.3 (21.4) months vs 64.1 (19.5) months; β = −5.88; 95% CI −14.1, 2.29] with no difference in attained mean (SD) height at mid-teen visit [165.2 cm (5.5) vs 165.3 (6.4); β = −.11, 95% CI −2.62, 2.40] (Table 2). Additionally, among participants with BMI <85th percentile, those with PCOS vs without PCOS had greater fasting insulin (β = 3.41, 95% CI 0.97, 5.84), HOMA-IR (β = .77, 95% CI 0.23, 1.30), and lower adiponectin/leptin ratio (β = −.35, 95% CI −0.65, −0.06) (Table 3). They also tended to have higher leptin and lower adiponectin, but the 95% CI crossed the null. We found similar results in our sensitivity analyses excluding PCOS cases based solely on menstrual irregularities and acne criteria (n = 3).

We observed a similar strength of association between adipokines and BMI in adolescent females with (n = 36) and without (n = 226) PCOS, including a strong positive association for leptin, (r = 0.71 and r = 0.75, respectively), a moderate negative association for adiponectin (r = −0.26, and r = −0.36, respectively), and a strong negative association for adiponectin/leptin ratio (r = −0.65, and r = −0.74, respectively).

**Figure 2.**
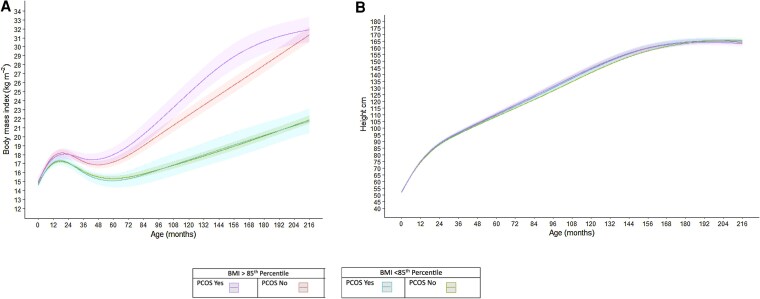
Estimated BMI (A) and linear growth trajectories (B) from birth through mid-teen visit, stratified by PCOS Status and BMI category. Abbreviations: BMI, body mass index; PCOS, polycystic ovary syndrome.

Figure 2 illustrates estimated BMI and linear growth trajectories from birth through the mid-teen visit by probable PCOS status (yes vs no) and BMI category (BMI ≥85th percentile vs BMI < 85th percentile). On visual inspection, BMI (kg/m^2^) trajectory was similar among those with BMI < 85th percentile among those with and without PCOS. Among those with BMI ≥85th percentile, however, participants with PCOS appear to have greater acceleration in BMI increase throughout childhood through the early teen years despite similar BMI by age 216 months (18 years old).

## Discussion

In this analysis of 358 adolescent females within the Project Viva prospective cohort, we assessed differences in growth trajectories and cardiometabolic biomarkers by PCOS status, stratified by BMI (≥85th and <85th percentile). We identified earlier age at peak height velocity, greater insulin resistance as measured by HOMA-IR, and an adverse adipokine profile among individuals with vs without PCOS among those with BMI <85th percentile but not when comparing PCOS status (with vs without) for participants within the BMI ≥85th percentile stratum. These findings are notable as they reveal that the cardiometabolic risk associated with PCOS exists even in individuals without an elevated BMI while also supporting the hypothesis of heterogeneity among individuals with PCOS (27, 28).

We observed differences in adipose tissue biomarkers among those with vs without PCOS within the BMI <85th percentile stratum. Among these “lean” participants, girls with PCOS had lower adiponectin/leptin ratio, indicating increased cardiometabolic risk and suggestive of adipose tissue dysfunction, compared to those without PCOS. While a more extensive literature base supports the adiponectin/leptin ratio as an established cardiometabolic risk factor among adults, a growing body of research supports its predictive utility in pediatric populations. Cross-sectional studies demonstrate that lower adiponectin and greater leptin levels in youth are a biomarker of metabolic syndrome risk (29, 30). From a longitudinal perspective, individual trajectories of adipokines may predict metabolic risk, with increasing leptin concentrations in early childhood associated with later adiposity (31). Prior findings in Project Viva align with this as well, as increasing leptin concentrations from birth through childhood were associated with greater adiposity in early teens in comparison with those with stable or decreasing leptin concentrations (32). Specifically regarding the adiponectin/leptin ratio, adolescents whose ratios decreased over time had less favorable cardiometabolic risk profiles compared to those with stable or increasing ratios (33). Additional research has explored the potential predictive finding of the adiponectin/leptin ratio in pediatric populations, including its value in distinguishing between type 1 and type 2 diabetes (34), the risk of weight gain among girls with central precocious puberty (35), and lower adiponectin levels independent of BMI in prepubertal daughters of women with PCOS (36). The differential predictive value for adiponectin/leptin ratio for adolescents vs adults is a gap in the literature that future studies should address.

Adipose tissue dysfunction, including dysregulated adipokine secretion, is hypothesized to be a key component of PCOS pathophysiology and is well described within the adult literature, in the context that PCOS is more frequently diagnosed among those with higher BMI (37). While adults with PCOS are observed to have greater leptin concentrations as well as an adverse adiponectin/leptin ratio that is associated with cardiometabolic risk (38), an adverse adipokine profile has also been described in nonobese women with PCOS (39).

In our current understanding of PCOS pathophysiology, questions remain regarding the interplay of adipokines with genetic drivers and androgen hormones. Polymorphisms in the *ADIPOQ* gene are nominally associated with risk of PCOS, suggesting that adiponectin may play a causal role (40); however, this finding has not been consistently identified across different populations in genome-wide association studies (41). In work examining the use of the adiponectin/leptin ratio as a predictive marker of PCOS in adult women, there was a weak albeit statistically significant negative correlation between testosterone concentrations and adiponectin/leptin ratio (−0.153; *P* < .05) (38). Additionally, limited data shows an inverse relationship testosterone and leptin concentrations among adult women with PCOS although the mechanism within PCOS is uncertain (42).

In identifying signs of adipose tissue dysfunction among adolescents with PCOS and BMI <85th percentile, the current study suggests that altered adipokine profile suggestive of adipose tissue dysfunction occurs in individuals with PCOS, even in the absence of clinical obesity. However, the presence of PCOS does not appear to modify the extent to which adipokines correlate to body mass. Although “lean” PCOS is hypothesized to be driven by neuroendocrine factors as opposed to interactions between adiposity and insulin resistance that are thought to be more characteristic of PCOS with obesity (27, 43), our findings highlight that adipose tissue dysfunction and associated insulin resistance are components of PCOS pathophysiology regardless of BMI category. These findings align with what we previously found during midlife in an analysis among adult participants of Project Viva, in which women with PCOS had an adverse adipokine profile, even after adjusting for BMI (44). Additionally, women meeting PCOS diagnostic criteria but lacking a formal self-reported diagnostic (termed “probable” PCOS) were characterized by lower BMI than those with “diagnosed” PCOS. However, those with “probable” PCOS had increased HbA1c and lower adiponectin concentrations vs women without PCOS characteristics.

Within this study, adolescents with BMI <85th percentile and PCOS also had higher insulin resistance compared to peers without PCOS. These findings build on prior work describing abnormalities in glucose tolerance in adolescents with PCOS in clinical samples. Among 66 adolescents with PCOS recruited from a multispecialty PCOS clinic, Flannery et al found impaired glucose tolerance following an oral glucose tolerance test that was present in adolescents characterized as both “lean” and “obese” PCOS (5). Palmert et al had similar findings, noting an approximately 1 in 3 prevalence of abnormal glucose tolerance among adolescents with PCOS (n = 27), with impaired glucose tolerance found among those with both “lean” and “obese” PCOS (6). In this work, authors posit that, even in the setting of normal fasting insulin and fasting glucose concentrations, impaired glucose tolerance is evidence of greater peripheral insulin resistance that may represent some of the first signs of the adverse metabolic consequences associated with PCOS.

These findings are important to consider as risk for weight gain and metabolic disease accumulate throughout the life course (45), and the population of females with PCOS and BMI <85th percentile may represent an at-risk group potentially in need of increased screening and recognition to mitigate the cardiometabolic risk of PCOS, as current pediatric guidelines focus on cardiometabolic screening among adolescents with BMI ≥85th percentile (26). Additional research from the Northern Finland Birth Cohort supports the importance of optimizing weight maintenance throughout young adulthood, as excessive weight gain between the ages of 14 to 31 years was associated with greater risk of later type 2 diabetes by the age of 46 among people with PCOS than those with PCOS without rapid weight gain (46)

Additionally, among those with BMI <85th percentile, we observed a younger age at peak height velocity among participants with vs without PCOS. This may potentially represent an effect of premature adrenarche—a proposed risk factor for PCOS—as children with premature adrenarche may have accelerated linear growth and pubertal development compared to those without premature adrenarche (47, 48). The physiology underlying premature adrenarche and earlier pubertal development in relationship to PCOS may be driving our finding of differences in growth velocity and pubertal timing, specifically among participants with PCOS and BMI <85th percentile. Prior work among prospective birth cohorts (in which this cohort was included) found an association between PCOS polygenic risk score and both younger age at pubarche and peak height velocity (49). Based on our current findings, this association may be driven by those with PCOS and BMI <85th percentile, as we did not observe this association among those with PCOS and BMI ≥85th percentile.

In contrast, among those with BMI **>**85th percentile, participants with PCOS had a similar metabolic profile and linear growth trajectory to those without PCOS. These findings suggest that, among adolescents in this sample, PCOS is not conferring additional metabolic risk beyond that which is associated with excess adiposity. This aligns with findings from Rossi et al, in which PCOS did not confer additional cardiometabolic risk to adolescents with obesity when compared to BMI-matched controls (50). However, these findings emphasize the importance of considering the comparison population in studies assess the contribution of PCOS to metabolic risk. In a case-control study in which girls with PCOS were compared to the general population through age- and ethnicity-matched participants of the Third National Health and Nutrition Examination Survey, metabolic syndrome risk increased even after adjusting for BMI and insulin resistance, a finding that may be overlooked when only studying PCOS in the setting of overweight and obesity (4).

Our findings add to the limited literature supporting that growth patterns may provide evidence of PCOS risk before the clinical phenotype is apparent. Within the Northern Finland Birth Cohort, lower birthweight, earlier adiposity rebound, and greater mean BMI throughout childhood were risk factors for later PCOS assessed at age 46 (12). A single study using the Copenhagen School Health Records Register along with the Danish National Patient Registry examined both BMI and linear growth, finding that both females with overweight and those with taller stature at ages 7 and 13 had a greater risk of later PCOS in adulthood (15). Putting our findings in this context, we similarly observed that childhood growth patterns may deviate among those with later PCOS. However, our findings add additional complexity to this observation and highlight the heterogeneity of the interplay between PCOS risk and growth, which may vary depending on adiposity status. While our sample size precludes predictive analyses, the early pubertal years may be a key period for assessing accelerated weight gain as a risk factor for later PCOS among adolescents with obesity whereas the prepubertal years may be characterized by earlier linear growth acceleration and a trend toward earlier adiposity rebound among those with PCOS risk without obesity.

## Strengths and Limitations

A primary strength of our work is its characterization of PCOS among individuals both with and without overweight/obesity, specifically in relation to cardiometabolic biomarkers, growth, and adiposity. Most data on cardiometabolic associations of PCOS in adolescents come from clinical samples of adolescents with PCOS. However, findings in the adult literature have shown that referral biases may impact both the severity and the clinical presentation of PCOS when comparing findings in clinical referral vs unselected populations (19, 20). Adolescents with obesity and PCOS characteristics are more likely to have a diagnosis code for PCOS within the electronic health record vs those without obesity who have PCOS characteristics (51). As a result, clinical populations of adolescents already with a PCOS diagnosis may both overstate the association between PCOS and adiposity as well as represent a more severe cardiometabolic phenotype than the overall population burden. Additionally, we had access to a variety of DXA measures of adiposity at the mid-teen visit, allowing assessment of body composition beyond BMI in association with PCOS status. The use of a prospective cohort also allows for longitudinal assessment of growth parameters over time before the appearance of PCOS later in adolescence.

A primary limitation of this work is the loss to follow-up over time, which may occur with any prospective cohort study. Our classification of PCOS relied on self-reports of menstrual cycle, physician diagnosis, and clinical hyperandrogenism, which may be less accurate than direct clinician assessment. Biochemical hyperandrogenism was assessed using metabolomic DHEA-S, which was available among participants, and not the gold-standard testosterone assays (2). However, sensitivity analyses performed as part our prior publications demonstrated our definition of probable PCOS was robust (16), similarly to the current study sensitivity analyses excluding adolescent classified as PCOS only on basis of acne + irregular menses showing similar results to our main analyses. We used mixed effects models calculate age at adiposity rebound and peak height velocity, as well as statistical approximations to provide a visual representation of BMI and height increases throughout childhood. However, this method has some limitations as it approximates the average growth trajectory in the population instead of an individual child's progression. Additionally, our identified growth patterns are descriptive and not meant to imply causality; various factors, including environmental exposures, genetic risk, and health behaviors, may influence growth and later PCOS status.

## Conclusion

In summary, we identified that PCOS status is characterized by adipose tissue dysfunction and insulin resistance in adolescents without overweight/obesity. Additionally, we observed that variations in earlier linear growth and adiposity trajectories are different by BMI category. This suggests that although PCOS may not present until later adolescence, it may be preceded by distinct growth patterns that may be associated with later PCOS risk. These findings highlight that the metabolic risk of PCOS is present throughout the BMI spectrum. Adolescents with PCOS, even with BMI within the normal range, may benefit from interventions that mitigate excess weight gain, which may exacerbate the metabolic associations among individuals with risk for PCOS.

## Data Availability

Some or all datasets generated during and/or analyzed during the current study are not publicly available but are available from the corresponding author on reasonable request.
